# Mapping out epistemic justice in the clinical space: using narrative techniques to affirm patients as knowers

**DOI:** 10.1186/s13010-021-00110-0

**Published:** 2021-10-26

**Authors:** Leah Teresa Rosen

**Affiliations:** Weill Cornell Medical College, New York, NY USA

**Keywords:** Epistemic injustice, Narrative, Patient-centered care, Race, Gender

## Abstract

Epistemic injustice sits at the intersection of ethics, epistemology, and social justice. Generally, this philosophical term describes when a person is wrongfully discredited as a knower; and within the clinical space, epistemic injustice is the underlying reason that some patient testimonies are valued above others. The following essay seeks to connect patterns of social prejudice to the clinical realm in the United States: illustrating how factors such as race, gender identity, and socioeconomic status influence epistemic credence and associatively, the quality of healthcare a person receives.

After describing how epistemic injustice disproportionately harms already vulnerable patients, I propose a narrative therapy intervention. This intervention can help providers re-frame their relationships with patients, in such that they come to view patients as valuable sources of unique knowledge. Though I identify this intervention as a valuable step in addressing clinical epistemic injustice, I call upon medical educators and practitioners to further uplift the voices, perspectives, and stories of marginalized patients.

## What is Epistemic Injustice?

Before imagining a path towards epistemic justice, one must first think critically about the ways in which epistemic *injustice* lives within our systems and society in the United States. And though the term itself may seem unfamiliar, I promise that its effects are not. Namely, this particular form of injustice was borne within, and since sustained through, our interpersonal and social realms. So in the same way that we have each witnessed or experienced racial, gender, or socioeconomic injustice in casual, professional, and private settings, epistemic injustice affects everyone, everywhere. Moreover, while the aforementioned injustices are perpetrated on the basis of race, gender, and socioeconomic status respectively, epistemic injustice pertains to epistemology—the theory of knowledge—and thus involves the wrongful discrediting of someone’s capacity as a knower [7].

Further, because knowledge is so intertwined with power in the modern Western world, epistemic injustice has become a device of domestic sociopolitical oppression in the United States: serving to frame already powerful persons as legitimate knowers, and in turn, debase the testimonies offered by persons with less power or social capital. To clarify, the following, highly popularized instances from recent years are each examples of epistemic injustice in action: the murder of George Floyd, the events prompting the #MeToo movement, and the incarceration of The Exonerated 5. Epistemic injustice is the force which renders some voices less credible than others. So when George Floyd cried, “I didn’t do nothing,” epistemic injustice is why his words were received with disbelief rather than respect [15]. And when a victim of sexual assault or harassment had the courage to share her story, epistemic injustice is the reason the story was subjected to scrutiny and criticism [10]. Finally, when five young boys claimed that they were innocent, epistemic injustice made it so their collective voices could not be heard over the single voice of an educated, white lawyer or defendant [2].

As evinced through these examples, any discussion of epistemic injustice cannot be separated from the wider discourse on race, gender, and identity discrimination; for, one’s social identity is shaped by each of these qualities in a unique and meaningful way. Therefore, intersectionality is an important concept to understand when evaluating the effects of epistemic injustice on different persons. Intersectionality, as defined by Patricia Hill Collins—an expert on black feminist thought and the politics of identity and empowerment—“references the crucial insight that race, class, gender, sexuality, ethnicity, nation, ability, and age operate not as unitary, mutually exclusive entities, but rather as reciprocally constructing phenomena that in turn shape complex social inequalities” ([4], p. 115). By this logic, a given black woman would be subjected to epistemic injustice not as a black person and a woman separately; but as a black woman wholly, whose identity is shaped by the unique and complex interactions of those qualities.

Then depending on how one’s various identity qualities overlap, different challenges may arise that interfere with one’s ability to earn an education or become employed. Insofar as education and employment status are often considered to be markers of intelligence or social status, the cycle continues whereby individuals born to less privileged circumstances are then treated as less credible knowers. And that is how epistemic injustice has hi-jacked our social world: making it so individuals with marginalized identity traits suffer oppression for the ways in which they are different, and then the oppression precludes them from having the opportunity to articulate or relay their experiences.

## Epistemic Injustice in the Clinical Realm

Medical institutions are not protected from the biases and prejudices of the outside world. If anything, the clinical realm reflects the injustices and inequalities that are present in US society, and can also further reinforce these issues by treating patients differently depending on their identity traits. Differential clinical treatment on the basis of race, gender identity, and socioeconomic status—though admonished in hospital mission statements and diversity declarations—is manifested through the limited access to equitable healthcare available to BIPOC, gender non-conforming, or low-income patients. Associatively, these specific patient groups are among the most likely to experience epistemic injustice during a clinical encounter [5].

In considering the treatment of BIPOC patients in the United States, there is extensive literature pointing towards racial bias in pain assessment and treatment [1, 9]. This could be because pain, like other subjective symptoms or disorders, cannot always be seen. Therefore, a patient’s testimony offers crucial clinical evidence, and if that testimony were to not be honored fully by the clinician, then the patient’s pain may not be treated fully. Unfortunately, this is a common outcome, as reflected in clinical data showing that pain experienced by black patients in the United States goes under-diagnosed and under-treated, when compared to pain experienced by white patients [14]. Similarly, Native Americans patients feel as if their voiced concerns are also going unheard. In the aptly-titled *New York Times *piece, “Fed up with deaths, Native Americans want to run their own health care,” Native American communities cite the numerous ways in which their testimonies have been ignored or overlooked, presumably because their identities, needs, and circumstances are different from those of other, more privileged patients [17]. This sentiment to some extent echoes the struggle of gender non-conforming patients, who have difficulty receiving equitable healthcare because the traditional medical realm lacks the hermeneutical resources to support and care for them [12]. And finally, low-income patients may experience epistemic injustice through forced opportunity cost due to restricted healthcare access; if a person is not able to afford insurance or a healthcare visit, she is not granted equal opportunity to share her story with a clinician.

Beyond these considerations, there are additional identity characterizations that are unique to the clinical realm that make a patient vulnerable to experiencing epistemic injustice. The first of these identities is as the “patient” within the “patient-provider” relationship, and the second—if applicable—is as the “ill person.” As a patient, a person is now situated into yet another power dynamic within the clinical space, overlapping with any pre-existing, still-present power relations. For instance, if I—a Latina woman—were to enter into a clinical exam room and discover that my physician identifies as a white man, then there could in theory be a power tension between us on at least three points: ethnicity, gender, and role. That is to say, the power dynamics of ethnicity and gender do not disappear when I engage with the physician as a patient. Rather, just as Collins’ definition of intersectionality would connote, they overlap in such a way with my now-salient patient identity and together create a new meaning. But because each of this hypothetical physician’s identity traits—i.e. white, man, professional—have a historical reputation of assuming superiority over others, I as the patient could be placed in a position of epistemic risk [4]. Though not necessarily, my testimony may be subjected to doubt or mistrust based on the negative stereotypes linked to my various identity traits. Specifically, as a woman, my complaints could be viewed as emotional or dramatic, and as a patient rather than a professional, I could be thought of as an unreliable source when describing how my body feels or functions. For each of these reasons—and countless more that do not apply to my personal situation, and which I could thus never fully understand—patients are placed in a position of epistemic fragility.

Perhaps some of the more epistemically fragile patients are those who are perceived as “ill persons.” Eleanor Alexandra Byrne further describes this phenomenon in “Striking the balance with epistemic injustice in healthcare,” as follows: “Where ill people are negatively stereotyped, the speaker’s testimony might be unfairly dismissed, excluded or seen as less valuable than it would otherwise have been if they were not ill. It is only by virtue of being a member of the stereotyped group that their testimony is received and acted upon differently” [3]. Byrne then launches into a discussion about persons who suffer from Chronic Fatigue Syndrome/Myalgic Encephalomyelitis, exemplifying how one’s identity as an “ill person”—especially if there is stigmatization or stereotyping surrounding the condition—can lead to a patient being viewed as a less credible source of knowledge. I would extend this argument to include other stigmatized conditions beyond CFS/ME, including mental illness and women’s health conditions. Mental and women’s health are two particular domains of care that remain undervalued in the clinical realm, an issue that is directly intertwined with epistemic injustice [8, 11]. If individuals who suffer from mental or women’s health conditions are viewed as “ill persons,” their insights may not be as highly valued in the clinical space. And if this is the case, there would be less of an opportunity for these patients to share their whole stories, and perhaps then their insights would not be used as a resource to improve the clinical treatment of these conditions.

## Forging Epistemic Justice

In imagining a path forward—one that seeks to make clinical epistemic injustice in the US less common—it begins with US healthcare providers fortifying their commitment to honor the voice of the patient. That is, every patient: regardless of racial background, gender identity, socioeconomic status, and other identity qualities. For if patients from minority groups were better embraced in the clinical encounter as valued contributors with embodied testimonies, then perhaps providers could better piece together the complex and important circumstances of each patient's life and provide more comprehensive and equitable care.

To this end, I propose that Michael White’s work in narrative therapy be adapted into a clinical intervention designed to address and prevent epistemic injustice. White, who founded the Dulwich Centre in Australia and wrote the canonical texts on narrative practice, devoted much of his scholarly attention to the drafting of maps and other strategies to help clinicians practice narrative therapy [6, 19]. A specific narrative strategy used by White is called “Reauthoring Conversations,” through which the clinician tries to unveil a story that is not presently being told [18]. In White’s practice, as he worked most often with families during times of conflict, he would try to uncover a story that highlighted positive attributes of a person or a relationship. For instance, if the dominant narrative being told about a person was marked by negative identity conclusions—such as, “he is so stubborn,” or “he is an unloving partner”—White would prompt the group to identify times when that narrative did not truly align with that person’s behavior—including, moments when the person was not being stubborn or was actually being a loving partner. White then referred to these chosen moments as “Unique Outcomes” and spent the remainder of the conversation strengthening an alternative narrative about the person that would better align with the more positive “Unique Outcome.” The goal of this strategy is to transform the way in which another person is perceived, by first identifying the negative influence of the dominant narrative being told about that person and then shifting attention to an alternative, more positive conception of the person [19].

This narrative strategy is applicable to clinical instances of epistemic injustice because the act of “Reauthoring Conversations” can be utilized to uncover and elevate the patient’s voice and story. If done in such a way to mirror White’s work, a clinical intervention could be designed to reveal the patient’s “Unique Knowledge,” rather than to illuminate unique personality traits or actions. For instance, if an eighty-five year old woman were to walk into a patient visit and the physician held pre-conceived negative biases against female or elderly patients, he may be less likely to take her worries or complaints seriously—therefore, committing a distinctively epistemic wrong and also compromising the quality of care that patient receives. However, if there was an intervention that would force the clinician to pause and instead consider the unique knowledge that the patient possesses—knowledge that he himself cannot possibly access or attain—then perhaps he would be more inclined to listen to the patient’s testimony. In turn, by becoming aware of the patient’s “Unique Knowledge,” the healthcare provider can more effectively and compassionately attend to the health concerns of the patient. Likewise, by feeling like her voice is valued and respected by the physician, the patient will likely feel more comfortable being engaged and cooperative in the clinical space. The intervention would, therefore, benefit both the physician and the patient and ultimately improve health outcomes of patients—particularly those who are most vulnerable to experiencing epistemic injustice.

When thinking about what this intervention would look like, I imagine it taking the form of a two-way conversation between a clinician and patient before the actual clinical visit. Given the nature of the added time commitment, this intervention would be most realistic in non-emergency settings, where the patient and provider will maintain a longitudinal relationship. The conversation itself can take as long as the involved parties feel is appropriate, but should last a minimum of 10 min. Though it is ideal for the conversation to be held in-person, where the patient and clinician can view each other’s body language and social cues, it could also take place over a virtual video platform or a standard telephone call. The following guides list a few example questions each person could ask during the conversation in order to uncover each other’s “Unique Knowledge” and henceforth establish a space of epistemic equity:

**Table:** 

Conversation Questions for Clinician to Ask Patient
1.What would you like me to know about you?
2.How would you describe your identity?
3.What have you wanted your clinicians to do better in the past?
4.How can I best demonstrate to you that I value and respect what you have to say?

**Table:** 

Conversation Questions for Patient to Ask Clinician
1.I have the following fears about how I might be treated in the clinical realm: ______. What do you make of these fears?
2.I value ______ in a relationship with a clinician. Will [given value] be present in our clinical encounters?
3.Apart from your medical expertise, what unique knowledge are you bringing to our encounter?

The next pair of guides are for reflection rather than conversation. The patient should complete the reflection questions before speaking with the clinician, as a way to prepare her responses. The clinician, on the other hand, should wait to reflect only after having the initial conversation with the patient, and can do so before the clinical visit as a reminder to honor the patient’s testimony.

**Table:** 

Reflection Questions for the Patient (Before initial conversation)
1.Do I have any intersectional identities?
2.Do I have any fears about how I will be treated because of my identity?
3.I am the world’s expert in _________
4.How do I know when I’m being disrespected? How do I know when I’m being respected?
5.What do I most value in a relationship with a clinician?

**Table:** 

Reflection Questions for the Clinician (After initial conversation; before clinical visit)
1.What unique knowledge can the patient contribute to my clinical understanding of her life and body?
2.How do I provide a space for her to share that knowledge?
3.How can I demonstrate that I respect what the patient is saying?

The conversation might only occur once, but the patient and clinician should re-visit the Reflection Questions as a tool of general introspective utility. If the intervention works out as intended, the clinician and patient would be entering the clinical office visit with a foundational understanding of one another’s identity and values, as well as an appreciation for each other’s unique knowledge. Not only does this foster trust within the clinical encounter and benefit the patient-provider relationship, the intervention also paves the way for epistemic justice in healthcare—which could ultimately benefit the wider community’s and population’s health [13].

For one, by creating the space for patients to reflect on and share their unique knowledge, providers can learn more about the ways in which medicine and other institutions oppress certain groups by blindly following normative, often exclusionary, practices. This is a widely useful lesson, as the first-person narratives of oppressed or marginalized persons can provide telling insights into silos of care and other means through which injustice is spread. Feminist philosopher Nancy Tuana expands on this idea by rationalizing, “Those who do the devalued work in a society, or who are oppressed and exploited in other ways, can learn how to use their oppressed social position as a source of insight about how social relations work—insight unavailable or at least hard to come by within the conceptual frameworks of dominant institutions.” Tuana continues, “shifting attention to this neglected area of concern through the lens of the experiences of [oppressed persons] serves to transform both *who* knows and *what* is known” ([16], pp. 127–128).

Indeed, it is only through thinking critically about epistemic injustice and how it operates to silence the voices of marginalized persons that we can begin to work towards excavating those voices and unveiling their stories. For within these stories are truths that are not presently popularized within the US clinical realm or society at large; truths of prejudice, discrimination, oppression, and violence. Truths that could bring light to the issues that are being perpetuated through our institutions; and finally, truths that upon recognition could help disrupt the normalizing, epistemically-exclusive status quo.

## Conclusion

I view this narrative therapy intervention as a helpful, but incomplete first step towards actualizing epistemic justice in medicine. The intervention itself is useless until medical providers, educators, and administrators acknowledge the role that the healthcare realm has played in excluding different types of knowledge and consequently, oppressing various types of people. I urge practitioners to consider the ways in which they have neglected certain patient testimonies and not others, and think critically about the forces—perhaps even subconscious ones—that motivated those decisions. Then I ask that practitioners think about the impact of these decisions, and how the decision pattern might align with the health disparities in our country. Were the requests of white patients honored more frequently than those of patients of color? Did patients with a graduate education speak in a way that was familiar to the provider, and therefore receive better quality care? Have complaints made by female patients been dismissed because of the negative stereotypes attached to the female gender identity?

Each of these are plausible, even common, occurrences in the clinical space that have legitimate, detrimental consequences for patients and their wider communities. After all, if epistemic injustice is not thoroughly and routinely addressed, then clinical decisions may continue to be made without equitable consideration of patient testimonies, and the effect of that bias will be more significant in communities of minority persons—thus disproportionately affecting already vulnerable populations of people.

I identify this intervention as a first step only because it is designed to be reactive rather than proactive. In order to bring about sustained epistemic justice in medicine, more widespread, proactive interventions should be instated during medical education and training, to complement current clinical efforts in ethics and patient-centered care. Medical, nursing, and even pre-health students should be introduced to the topic of epistemic injustice as early as possible, so they can begin to evaluate its impact in the world around them before even interacting with patients. If these students become attuned to the ways in which some testimonies are afforded credibility and others are not in the social world, then they can prepare to empower and uplift the voices that are often discredited. In time, I hope that advocacy of this type becomes an expected competency for incoming health professionals, as it is clearly vital to the practice of equitable, patient-centered care.

But until then, Michael White’s teachings on narrative therapy provide an innovative and pragmatic starting point to resolving epistemic injustice in the clinical realm of the United States—an issue that is by no means the norm, but still common and problematic. By engaging in a conversation and reflective exercise designed to highlight a patient’s “Unique Knowledge,” providers can begin to map out how to better honor a range of patient testimonies and therefore be more inclusive of diverse patient backgrounds. This would not only make the patient a more involved co-participant in her healthcare journey, it would broaden the provider’s understanding of sociocultural determinants of health and also contribute to the narrowing of health disparities. So in thinking about our hopeful pursuit towards epistemic justice, narrative therapy appears to be a valuable tool: one which guides us to recognize that some crucial narratives have been buried along the way, and then helps us to bring those stories and their tellers to the surface again—to he heard, and to be honored.

## Data Availability

Data sharing is not applicable to this article as no datasets were generated or analysed during the current study.

## Abbreviations

BIPOC: Black, indigenous, and people of color
BIPOC: Black, indigenous, and people of color
